# The relationship between maternal anxiety and cortisol during pregnancy and birth weight of chinese neonates

**DOI:** 10.1186/s12884-018-1798-x

**Published:** 2018-06-27

**Authors:** Fenling Fan, Yuliang Zou, Yushun Zhang, Xiancang Ma, Junbo Zhang, Cai Liu, Jie Li, Meili Pei, Yu Jiang, Anthony M. Dart

**Affiliations:** 1 Department of Cardiovascular Medicine, the First Affiliated Hospital of Medical College, Xi’an Jiaotong University, Xi’an, 710061 China; 2 Department of Gynaecology and Obstetrics, the First Affiliated Hospital of Medical College, Xi’an Jiaotong University, Xi’an, 710061 China; 3 Department of Psychology and Psychiatry, the First Affiliated Hospital of Medical College, Xi’an Jiaotong University, Xi’an, 710061 China; 4 Department of Cardiovascular Medicine, The Alfred, Baker Heart and Diabetes Institute, Melbourne, Vic 3004 Australia

**Keywords:** Pregnancy, Anxiety, Cortisol, Trimester, Birth weight

## Abstract

**Background:**

To determine the relationship between maternal anxiety and cortisol values and birth weight at various stages of pregnancy.

**Methods:**

Two hundred sixteen pregnant Chinese women were assessed for anxiety and depression and had measurement of morning fasting serum cortisol. Women were assessed either in the first (71), second (72) or third (73) trimester. Birth weights of all children were recorded.

**Results:**

There were significant negative correlations between anxiety level and birth weight of − 0.507 (*p* < 0.01) and − 0.275 (*p* < 0.05) in trimesters 1and 2. In trimester 3 the negative relation between anxiety and birth weight of −.209 failed to reach significance (*p* = 0.070). There was no relation between depression and birth weight in any trimester (*p* > 0.5 for all). Maternal cortisol was significantly inversely related to birth weight in trimester 1 (*r* = − 0.322) and with borderline significance in trimester 2 (*r* = − 0.229). Anxiety score and maternal cortisol were significantly correlated in each trimester (*r* = 0.551, 0.650, 0.537). When both anxiety score and maternal cortisol were simultaneously included in multiple regression analyses only anxiety score remained significant.

**Conclusion:**

Whilst both maternal anxiety score and maternal cortisol are inversely related to birth weight the associations with anxiety score were more robust perhaps indicating the importance of mechanisms other than, or in addition to, maternal cortisol in mediating the effects of anxiety. The findings indicate the importance of measures to reduce maternal anxiety, particularly of a severe degree, at all stages of pregnancy.

**Trial registration:**

The study was approved by the Ethics Committee of the 1st Affiliated Hospital of Xi’an Jiaotong University.

## Background

It is well recognised that low birth weight, generally defined as < 2.5 kg (Kg), is associated with deleterious effects in adult life, including increased susceptibility to cardiovascular disease [1–4]. In addition it has been shown that even in the ‘normal’ birth weight range inverse relationships exist between birth weight and a range of health outcomes such as blood [5] pressure. Thus aside from any immediate consequences of variation in birth weight, understanding the causes of this variation are of importance to health in later life.

A number of factors are known to affect birth weight [6]. These include maternal cigarette smoking, maternal nutrition, foetal abnormalities, maternal diabetes and others. Psychological disturbances are common during pregnancy and can include features of both anxiety and depression. Estimates of their prevalence have varied widely probably depending in part on the nature of the instruments as well maternal background. Studies from the UK reported anxiety in 22% and depressive symptoms in 19% of women during pregnancy [7]. Other studies have reported rates of up to 60% for anxiety and 30% for depressive symptoms [8, 9]. A number of studies have examined the relationship between maternal psychological wellbeing and birth outcomes. A majority [10–14], but not all [15, 16] have reported that increased state anxiety in mothers, assessed using a variety of instruments, is associated with prematurity and/or low birth weight. Some studies have found relationships between birth outcomes and stressful life events but not state anxiety [17, 18]. The relationship between maternal state anxiety and distribution of birth weights in term infants has been less well studied with less consistent results [12]. Association between maternal depression and birth outcomes have also been reported [19].

We recently demonstrated an inverse relation between maternal circulating cortisol levels and birth weight [20]. Anxiety is a complex state with multiple physiological associations including changes in levels of stress hormones, activity of the autonomic system and effects on the cardiovascular system including uterine artery resistance [21]. A previous study using foetal biometry reported an inverse association between urinary cortisol and estimated birth weight [12]. Interestingly in this study anxiety state was not significant in multivariate analysis. We have here examined the associations between both anxiety and maternal cortisol and measured birth weight to investigate the extent to which the associations of birth weight with anxiety might be mediated by concomitant changes in maternal cortisol.

## Methods

Full details of the cohort have been given previously [20, 22]. In brief, levels of anxiety were assessed by questionnaire in 231 pregnant Chinese women attending a major university hospital for antenatal care. 15 women were lost to follow-up, leaving 216 included in the final data analysis. All gave birth in hospital and birth weight were obtained from hospital records. All women were in their first (only) pregnancy. Women were assessed at a single time point during the first (71), second (72) or third trimester (73). In addition, fasting blood was drawn from the women between 9 am and 11 am for measurement of serum cortisol. Pregnancy duration at time of cortisol measurements (weeks) were 8.4 ± 1.6, 20.9 ± 4.4 and 33.8 ± 3.1. The study was approved by the Ethics Committee, the 1st Affiliated Hospital of Xi’an Jiaotong University. Written informed consent was obtained.

### Assessment of anxiety & depression

Anxiety was assessed with The Hamilton Anxiety Scale (HAMA). A psychologist or trained physician asks 14 semi-structured questions, 7 related to psychological anxiety symptoms and 7 related to somatic anxiety symptoms, and then provides a score according to a 5-point scale for each item (0 - Not at all; 1 – Mild; 2 – Moderate; 3 – Severe; 4 - Very Severe). The total scores are used for assessment (Mild Anxiety: 18–25; Moderate Anxiety: 26–30; Severe Anxiety: > 30).

The HAMD is the most widely used outcome scale for depression studies. The 24-item HAMD questionnaire is the most common version used in mainland China scoring 5 points for each item as described for HAMA. The total scores are then used to quantify depression (Mild Depression: 10–19; Moderate Depression: 20–29; Severe Depression: > 30). The two scales were translated and rigorously tested with different populations in mainland China. Their reliability and validity were assessed and approved by the responsible authority (Shanghai Mental Health Center).

### Measurement of serum cortisol

Venous blood samples were taken between 9 and 11 am, in the fasting state. The samples were immediately put on ice and the obtained serum was frozen at − 80° centigrade (C). Samples were subsequently thawed for determination of cortisol levels by radioimmunoassay (Jiuding Biotechnology, Tianjin, China) at the hospital chemical pathology department and in accordance with the manufacturer’s instructions. Coefficient of variation within each batch was < 5%.

### Statistical analysis

Statistical analysis was undertaken with SPSS v19. Group data is presented as mean ± sd. Associations between variables were assessed using Spearman correlation coefficient for correlations involving levels of anxiety and Pearson correlation coefficient for other associations. Multiple regression analysis was performed using stepped entry and removal. Analysis was conducted separately for each trimester to evaluate whether effects of anxiety or cortisol were trimester dependent.

## Results

Mothers were aged 26.4 ± 3.2 years, had pre-pregnancy weight of 53.4 ± 4.0 Kg and height of 160 ± 3.6 cm (cm). All mothers were in their first (only) pregnancy and were from metropolitan areas. All were of Han Chinese ethnicity and gave birth in the first affiliated hospital of Xi’an Jiaotong University. None of the mothers drank alcohol or smoked cigarettes during the pregnancy (and only 4 reported smoking prior to pregnancy), none were diabetic or hypertensive. All pregnancies proceeded to term. Birth weights of the children were 3.25 ± 0.47, 3.36 ± 0.33 and 3.43 ± 0.38 Kg, for those whose mothers were recruited in the first, second and third trimester respectively. There were no significant correlations between pre-pregnancy maternal weight and childrens birth weight (− 0.078) nor between maternal height and childrens birth weight (− 0.082).

**Fig. 1:**
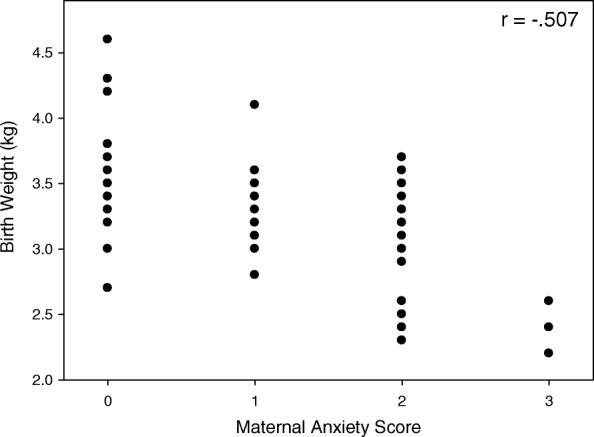
The figure shows the relationship between maternal anxiety score and birth weight for mothers recruited in the first trimester. r Spearman rank coefficient

**Fig. 2:**
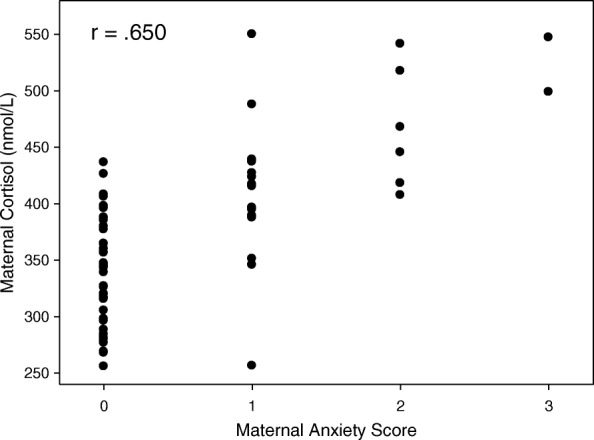
The figure shows the relationship between maternal anxiety score and maternal cortisol for mothers recruited in the second trimester. r Spearman rank correlation

Mild, moderate and severely elevated anxiety scores were present in 30.9, 29.5 and 5.6% of mothers recruited in trimester 1, 22.2, 8.3 and 2.7% of mothers recruited in trimester 2 and 32.8, 6.8% and 0% of mothers recruited in trimester 3. Respective rates for anxiety of any degree were 66.0%, 33.2 and 39.6%. Depression of any degree was found in 47, 54.1 and 47.9% for trimesters 1, 2 and 3 respectively. Moderate or severe depression was present in 12.6%, 11.1 and 8.2% of trimester 1, 2 and 3 respectively. Whilst 33.4, 23.6 and 23.2% were found to have both anxiety and depression (of any degree). There were significant negative correlations between anxiety level and birth weight of − 0.507 (*p* < 0.01) and − 0.275 (*p* < 0.05) in trimesters 1 (Fig. 1) and 2. In trimester 3 the negative relation between anxiety and birth weight of −.209 failed to reach significance (*p* = 0.070). However, there were no significant correlations between levels of depression and birth weight in any trimester(*p* > 0.5 for all).

Maternal cortisol values were 341 ± 85, 371 ± 66 and 411 ± 7 nmoles/litre (nmol/l) in trimesters 1, 2 and 3 respectively. In order to examine whether the effects of anxiety could be mediated by changes in maternal cortisol we firstly examined the dependence of birth weight on cortisol. As seen in Table 1 there were significant correlations between birth weight and cortisol in the first trimester and borderline significant correlations in the second but not in the third trimester. We then examined whether changes in cortisol were related to levels of anxiety (Table 2, Fig. 2) which was significant for all trimesters.

**Table 1:** The Table shows Pearson correlation coefficients (r) between birth weight and maternal serum cortisol separately for each trimester. Respective p values are shown in brackets

	Maternal Cortisol (nmol/l)		
	Trimester 1	Trimester 2	Trimester 3
Birth Weight (Kg)	−0.322 (*p* = 0.006)	−0.229 (0.053)	− 0.192 (0.103)

**Table 2:** The Table shows Spearman rank correlation coefficients (r) between maternal cortisol and anxiety score, separately for each trimester. Respective *p* values are shown in brackets

	Maternal Cortisol (nmol/l)		
	Trimester 1	Trimester 2	Trimester 3
Maternal Anxiety Score	0.551 (*p* < 0.001)	0.650 (*p* < 0.001)	0.537 (*p* < 0.001)

We then undertook multiple analysis separately for each trimester with birth weight as the dependant variable and including anxiety level, depression level, maternal cortisol, maternal height and maternal weight as independent variable. In these analyses maternal anxiety was the only significant term with all other variables having *p* > 0.5 (Table 3).

**Table 3:** The table shows results from multiple regression analyses with birth weight as the dependent variable and including maternal anxiety, maternal depression, maternal cortisol, maternal height, maternal weight as independent variables

Anxiety					
	Overall r	Unstandardised Coefficient		Standardised Coefficient	*p*
		B	SEM		
Trimester 1	.591	−.300	.049	−.591	< 0.001
Trimester 2	.355	−.154	.049	−.365	0.002
Trimester 3	.373	−.228	.067	−.373	< 0.001

Analysis used stepped entry (PIN 0.05) and removal (POUT 0.1). Anxiety was the only significant independent variable in each of the trimesters. Respective significance values (p) for cortisol were .895, .804 and .887 in trimesters 1, 2 and 3 respectively

## Discussion

This study confirms an inverse relation between maternal anxiety and birth weight. As previously reported there was also an inverse relation between maternal cortisol and birth weight [20]. The effects of anxiety and maternal cortisol were more evident in early compared with late pregnancy. This is the first study in which the effects of maternal anxiety and circulating cortisol on measured birth weight were evaluated in the same mothers. As expected there was a strong correlation between anxiety level and circulating cortisol.

The significant association between maternal anxiety and maternal cortisol combined with the association between maternal cortisol and birth weight is compatible with the notion that the effects of anxiety on birth weight are mediated through maternal cortisol in the first and possibly also in the second trimester. However, when both parameters were included in multiple regression analyses only the effects of maternal anxiety remained significant. Whilst this may be an indication that factors other than maternal cortisol mediate (or contribute to) the link between maternal anxiety and birth weight, it might also be an indication that the assessment by questionnaire of anxiety at one time point during pregnancy is a better reflection of the prevailing psychological status of the mother than a single cortisol assessment is of the prevailing level of cortisol. In contrast to the current findings Diego et al. found that urinary cortisol but not anxiety state was predictive of estimated (fetal biometry) birth weight when both were entered into multivariate analysis. Interestingly this study was conducted in the second trimester when anxiety is less prevalent [21, 23]. Depression was also common in this cohort but there was no relation between depression and birth weight.

Whilst maternal corticosteroids are subject to the effects of placental 11β hydroxysteroid dehydrogenase type 2 it is known that there is sufficient trans placental transfer of cortisol to affect foetal growth and physiology. Thus hydrocortisone infusion during pregnancy in sheep affects not only foetal growth but also foetal cardiac parameters and blood pressure [24]. Human studies have also reported effects of maternal cortisol on infant neuro and cognitive development [25–28]. Previous studies have indicated that although the placenta itself may be a source of ACTH like peptides maternal ACTH does not appear to access the foetal circulation [29–32].

Other concomitants of heightened anxiety, such as alterations in the maternal autonomic nervous system, might also contribute to the effects of anxiety. Catecholamines in the maternal circulation seem unlikely to access the foetal circulation given the evidence that placental clearance of catecholamines is extensive with significant activity of catechol-O-methyl transferase and monoamine oxidase activity present in placental tissue [33–35]. In addition the placenta is known to contain the noradrenaline uptake transporter [36]. However, there may be effects on placental vascular function leading to impaired foetal growth. Changes in maternal haemodynamic parameters associated with stress may also be relevant. Anxiety in the non-pregnant state has also been associated with a range of other effects including on immune function, circulating cytokines and other hormones which may affect placental function [37–41].

Birth weight is known to be affected by a number of factors in addition to anxiety and it is possible that the effects of anxiety are also mediated by one or more of these [6]. For example, anxiety may be expected to influence maternal nutrition which is a well-recognised factor influencing birth weight. Whilst cigarette smoking is a potent influence on birth weight and will likely be affected by anxiety maternal cigarette smoking will not be a factor in the current study as none of the pregnant women smoked cigarettes during the pregnancy (and indeed very few were cigarette smokers prior to pregnancy). The mothers could have been exposed to second hand cigarette smoke given the high prevalence of cigarette smoking among Chinese males [42] but this was not quantified in the current study. Effects on maternal caffeine and alcohol intake are also not likely to be responsible given the very low consumption of coffee amongst Chinese women and the avoidance of alcohol during pregnancy.

The current study cannot determine whether the anxiety scores determined during pregnancy were representative of the non-pregnant state or were predominantly related to pregnancy as anxiety scores were not available in the mothers at other times. Estimates of the prevalence of anxiety and depression during pregnancy in other cohorts have varied. In a study from the UK anxiety was found in approximately 22% and depression in 19% of women during pregnancy [7]. Studies from Finland and South America have reported anxiety rates of between 16 and 60% [8, 9] whilst overall rates of psychosocial stress 40–84% have been reported amongst pregnant Chinese women [43]. In addition to ethnic or cultural factors these differences most likely also relate to differences in the instruments used to detect and quantify anxiety and depression. However, all studies find anxiety to be common during pregnancy and more prevalent than for women in the non-pregnant state.

Variation in levels of anxiety during pregnancy has also been reported with higher prevalence’s in the 1st and 3rd trimesters [23, 44]. In agreement with the findings reported here levels are higher in the 1st than the 3rd trimester in primigravidae.

## Conclusion

The findings from this, and other, studies suggest that measures to lessen anxiety during pregnancy may have an effect on birth weight and thus on the health of off-spring in later life. The effects of heightened anxiety appear not to be fully explained by changes in maternal cortisol.

## Abbreviations

C: Centigrade
cm: centimetres
Kg: Kilogram
nmol/L: nanomoles/litre
sd: Standard deviation
